# The Efficacy of Solanezumab in Patients with Alzheimer’s Disease: A Systematic Review and Meta-Analysis of Clinical Trials

**DOI:** 10.3390/pharmaceutics17080999

**Published:** 2025-07-31

**Authors:** Mathias S. Renteros, Renzo Barreto-Abanto, Diego C. Huapaya, Mateo Tovar-Cobos, Richard D. Alvarado-Ramos, Oriana Rivera-Lozada, Joshuan J. Barboza

**Affiliations:** 1 School of Medicine, Universidad Peruana de Ciencias Aplicadas, Lima 15023, Peru; mathias.renteros@gmail.com; 2 School of Medicine, Universidad Privada Antenor Orrego, Trujillo 13008, Peru; renzobarreto01@gmail.com; 3 School of Medicine, Universidad Ricardo Palma, Lima 15039, Peru; dalejandrohc2020@gmail.com; 4 School of Medicine, Universidad San Francisco de Quito, Quito 170901, Ecuador; mateotovarc1@gmail.com; 5 Faculty of Health Sciences, Universidad Católica Sedes Sapientiae, Rioja 22826, Peru; ridanielalvarado27@gmail.com; 6 Vicerrectorado de Investigación, Universidad Señor de Sipan, Chiclayo 14002, Peru; riveraoriana@uss.edu.pe; 7 School of Medicine, Universidad Señor de Sipan, Chiclayo 14002, Peru

**Keywords:** systematic review, mild cognitive impairment, disease-modifying therapies, randomized controlled trials, neurodegeneration, biomarker-guided treatment, placebo, treatment outcome

## Abstract

**Background/Objectives:** Solanezumab is a humanized monoclonal antibody designed to bind soluble amyloid-beta (Aβ) and facilitate its clearance from the brain, aiming to slow the progression of Alzheimer’s disease (AD). **Methods**: A systematic search was applied in four medical databases through October 2024 to identify phase 2 or 3 randomized controlled trials evaluating solanezumab in patients aged ≥50 years with mild AD or in preclinical stages. The primary outcomes were changes in cognitive and functional scales, including ADAS-cog14, MMSE, ADCS-ADL, and CDR-SB. Data were pooled using a random-effects model, and certainty of evidence was assessed using GRADE. **Results:** Seven trials involving 4181 participants were included. Solanezumab did not significantly reduce cognitive decline based on ADAS-cog14 (MD = −0.75; 95% CI: −2.65 to 1.15; very low certainty) or improve functional scores on ADCS-ADL (MD = 0.85; 95% CI: −1.86 to 3.56; very low certainty) and CDR-SB (MD = −0.15; 95% CI: −0.89 to 0.60; very low certainty). A modest but statistically significant improvement was observed in MMSE scores (MD = 0.59; 95% CI: 0.33 to 0.86; moderate certainty). **Conclusions:** While solanezumab may offer slight benefits in general cognitive performance, its overall impact on clinically meaningful outcomes remains limited. The results do not support its use as a disease-modifying therapy for Alzheimer’s disease in either preclinical or symptomatic stages.

## 1. Introduction

Research into Alzheimer’s disease has evolved substantially since its first clinical description in 1906. The identification of amyloid plaques and neurofibrillary tangles in the early 20th century laid the foundation for understanding its neuropathological hallmarks. In the 1980s, Glenner and Wong introduced the amyloid cascade hypothesis, positioning amyloid-beta as a central pathological agent [1]. Subsequent decades saw the development of tau-centered models, biomarker-based diagnostic criteria, and the emergence of neuroimaging tools [2]. Despite numerous clinical trials, most therapies targeting amyloid or tau have failed to show significant clinical benefit, with recent monoclonal antibodies (e.g., aducanumab and lecanemab) offering controversial and limited efficacy [3,4].

Alzheimer’s disease (AD), initially identified by Alois Alzheimer in 1906, represents a prominent neurodegenerative disorder and a primary cause of cognitive decline in the global population, posing an escalating threat to public health as the world’s population ages [5,6,7]. According to the Global Burden of Disease report, more than 55 million individuals currently live with dementia, and Alzheimer’s accounts for 60–70% of these cases [8]. The incidence of AD nearly doubles every 5 years after the age of 65, with the majority of patients being over 75 years old [9]. The burden of Alzheimer’s disease becomes especially apparent when considering its impact on older adults. Marked by progressive neuronal deterioration, it leads to a gradual and steady decline in memory and cognitive functions, including short-term memory loss, neuropsychiatric symptoms, and impaired executive function [10]. Said long-term neuronal dysfunction is caused by the accumulation of β-amyloid plaques (Aβ) in the extracellular space and neurofibrillary tangles of hyperphosphorylated tau [1,2]. This complex mechanism proposed as the “amyloid cascade hypothesis” in 1984 has been considered as a driver of Alzheimer’s pathological processes and the leading theory of AD pathogenesis [1,3].

The global economic costs of dementia increased from USD 279.6 billion in 2000 to USD 948 billion in 2016, with the highest burden in Europe and North America [10]. In primary care, early detection protocols rely on a combination of clinical history, caregiver-reported symptoms, and standardized cognitive assessments such as the Mini-Mental State Examination (MMSE) or the Montreal Cognitive Assessment (MoCA) [11,12]. Neuroimaging and cerebrospinal fluid biomarkers are often unavailable at the first level of care, underscoring the need for referral systems and access to specialized diagnostic services. Literature on dementia care infrastructure emphasizes the importance of multidisciplinary teams, trained general practitioners, memory clinics, and access to pharmacological and non-pharmacological interventions [13].

Despite nearly four decades since the introduction of the widely recognized amyloid cascade hypothesis, therapeutic advancements in AD remain limited. Current treatments aim to control symptoms and slow disease progression, primarily through cholinesterase inhibitors and NMDA receptor antagonists, which target the acetylcholine and glutamate neurotransmitter pathways implicated in AD pathogenesis [13]. Given the central role of amyloid-beta (Aβ) in disease onset, drug development efforts have focused on reducing its synthesis and accumulation. However, translating these pathological insights into effective therapies has proven challenging. Symptomatic treatments have offered only modest clinical benefits, prompting a shift toward disease-modifying strategies. These include targeting amyloid or tau pathology directly, such as through monoclonal antibodies like solanezumab [14].

From a pharmaceutical and biomolecular standpoint, therapeutic interventions in Alzheimer’s disease target several molecular pathways. Approved symptomatic treatments such as donepezil—a reversible acetylcholinesterase inhibitor—act by prolonging cholinergic neurotransmission and are chemically characterized by a benzylpiperidine-indanone scaffold [15]. In contrast, biological agents such as solanezumab are monoclonal antibodies that lack a traditional small-molecule structure. Solanezumab specifically binds to the central epitope of soluble amyloid-beta (Aβ1–42), facilitating peripheral clearance via immune-mediated pathways [16]. Although its complete protein structure cannot be fully represented by a classical chemical diagram, its mechanism of action has been thoroughly described in this manuscript to reinforce its pharmacological basis.

Solanezumab, a humanized monoclonal antibody, targets the middle domain of soluble amyloid-beta (Aβ). Its detection and removal from the brain are facilitated by binding to this peptide. By eliminating soluble amyloid beta and improving cognitive performance in Alzheimer’s patients, it is hypothesized that solanezumab may delay disease progression [6]. In the following years, several clinical trials were developed in response to these claims, providing evidence of solanezumab’s ability to modulate the disease. However, most of the trials that have evaluated this drug have yielded mixed results. The EXPEDITION-1 and EXPEDITION-2 trials used the Alzheimer’s Disease Rating Scale (ADCS-Cog11) and the Alzheimer’s Disease Cooperative Study Activities of Daily Living Scale (ADCS-ADL) to assess the drug’s efficacy in terms of cognitive outcomes. Neither study showed significant improvements in these outcomes after 18 months of treatment, with changes from baseline of less than 1 point [17].

Developing disease-modifying therapies for Alzheimer’s disease entails not only scientific complexity but also substantial economic and logistical challenges. Global investment in Alzheimer’s research exceeded USD 3.2 billion in 2023 alone, with significant contributions from public institutions such as the National Institutes of Health (NIH), private foundations, and the biopharmaceutical industry [11]. Clinical trials evaluating monoclonal antibodies like solanezumab often require multinational coordination, advanced biomarker infrastructure, and thousands of enrolled participants, resulting in estimated costs surpassing USD 100 million per phase 3 trial [16]. Additionally, the human resources involved—including clinicians, imaging specialists, data analysts, and regulatory personnel—highlight the enormous interdisciplinary effort needed to evaluate new treatments. Despite these investments, the high rate of trial failures underscores the need for improved therapeutic targets and strategic allocation of research funding [4,15].

In addition to established symptomatic treatments such as donepezil (a benzylpiperidine-based acetylcholinesterase inhibitor) and memantine (an NMDA receptor antagonist), recent therapeutic efforts have focused on anti-amyloid monoclonal antibodies, including aducanumab and lecanemab, which have received accelerated approval for early-stage Alzheimer’s disease [3,4]. Furthermore, several molecular classes are under active investigation as potential anti-Alzheimer agents. These include β-secretase (BACE1) inhibitors, γ-secretase modulators, tau aggregation inhibitors, and multitarget-directed ligands (MTDLs) designed to simultaneously modulate cholinergic, glutamatergic, and oxidative stress pathways [15]. Promising chemical scaffolds such as benzofurans, indole derivatives, and pyrimidine-based compounds are being explored for their capacity to cross the blood–brain barrier and interact with multiple pathological targets [6]. These innovations represent a shift toward more integrated pharmacological strategies aiming to alter the disease course beyond amyloid modulation.

The aim of this review is to compile data demonstrating the efficacy of solanezumab in improving cognitive outcomes in patients with Alzheimer’s disease.

## 2. Materials and Methods

This study was a systematic review with a meta-analysis. The reference elements for systematic reviews and meta-analyses (PRISMA-2020) informed this review. The PROSPERO registered was indexed in CRD420251025180.

### 2.1. Searches

Databases such as PubMed, Scopus, Web of Science, and EMBASE were used. Searches were performed from the inception until 4 October 2024, including key phrases, MESH (PubMed), and Emtree thesauri (Scopus, Embase). Finally, a search strategy was applied to each database (Supplemental Table S1). (“Alzheimer’s Disease”) AND (“Solanezumab”) were the primary search phrases. There were no restrictions on language or publication date. Additionally, all reference lists from relevant studies and included review articles were manually searched for other potentially eligible trials.

### 2.2. Eligibility Criteria

Inclusion Criteria: This systematic review included all studies that met the following criteria: Phase 2 or Phase 3 randomized controlled trials that treated patients over 50 diagnosed with mild Alzheimer’s disease, as defined by a Mini-Mental State Examination score of 15 to 26 points. Compared to a placebo, the intervention involved solanezumab (400 mg administered every 4 weeks for at least 18 months). Studies evaluating patients at risk for Alzheimer’s disease treated exclusively with a solanezumab intervention were also included.

Exclusion criteria: Conference abstracts, systematic reviews, narrative reviews, case reports, case series, and letters to the editor were excluded, as were randomized controlled trials that evaluated solanezumab in addition to another drug simultaneously in the treatment for Alzheimer’s.

### 2.3. Outcomes

The primary outcomes were disease progression measured through different cognitive scales. The ADAS-Cog14 questionnaire was a standardized tool that evaluated 14 components (word recall, naming objects and fingers, following commands, constructional praxis, ideational praxis, orientation, word recognition, remembering test instructions, spoken language ability, word-finding difficulty, comprehension, delayed word recall, number cancelation, and maze completion); designed to assess cognitive performance and deterioration in individuals with suspected or confirmed Alzheimer’s disease. Scores ranged from 0 to 90, where higher scores indicated more significant cognitive impairment. Another scale, the MMSE (Mini-Mental State Examination), was also evaluated for cognitive impairment and dysfunction using a 30-point-based system divided into five key areas: Orientation, registration, attention and calculation, recall, language, and praxis. Having a maximum score of 30, a value less than 25 reflected some cognitive impairment. Similarly, the caregiver-reported ADCS-ADL questionnaire assessed the patient’s ability to perform daily living activities like dressing, eating, managing finances, etc. With the score range being between 0 and 78, lower scores indicated a more significant functional decline. The final questionnaire instrument used was the CDR-SB (Clinical Dementia Rating-Sum of Boxes), a quantitative tool used to monitor the severity of dementia by estimating six domains: memory, orientation, judgment, and problem solving, community affairs, home and hobbies, and personal care. Each domain was scored based on patients’ cognitive and functional performance ranging from 0 to 18, with higher scores indicating severe impairment.

The secondary outcomes included amyloid levels evaluated by its concentration changes measured in serum and cerebrospinal fluid and solanezumab adverse events. The latter was defined as any adverse events reported in the intervention populations with a significantly increased frequency than in the control group.

### 2.4. Data Extraction

After the electronic searches, the results were compiled in a single library, and duplicates were eliminated. Then, independently and blinded, the first screening step was performed, evaluating the titles and abstracts and applying the inclusion and exclusion criteria to each result reviewed through the Rayyan platform. The studies included after this phase were searched and analyzed in full text, and then a new screening process was carried out, justifying the inclusion and exclusion criteria. After this process, the eligible studies were included in the systematic review, and data extraction began. A third review author (JJB) was consulted in case of disagreement. Data were extracted from each study individually and blinded using a pre-prepared Excel spreadsheet format. For each analysis, data were extracted on the author, year of publication, country, type of study, number of participants per intervention arm, selection criteria, description of intervention and control, and primary and secondary outcomes.

### 2.5. Risk of Bias Assessment

The risk of bias (RoB) was independently assessed using the RoB 2.0 tool. Disagreements were resolved by discussion with a third author (JJB). RoB per domain and study was described as low, with some concerns, and high for RCTs.

### 2.6. Data Synthesis

Random-effect models with the inverse variance method were used for all meta-analyses of the effects of solanezumab compared with placebo on primary and secondary outcomes. The Paule-Mandel method was used to calculate the between-study tau^2^ variance. The impact of solanezumab compared to placebo on dichotomous outcomes was described with relative risks (RR) and their 95% confidence intervals (CI). We adjusted for null events in one or two arms of the RCT using the continuity correction method. It was adjusted using the Hartung-Knapp method if more than five studies were found for meta-analysis. Statistical heterogeneity of effects among RCTs was described by the I^2^ statistic, whose values represented low (<30%), moderate (30–60%), and high (>60%) levels of heterogeneity. For sensitivity analysis, fixed effects and the Mantel-Haenszel method were used. The metabin function of the R 3.5.1 meta library (www.r-project.org; accessed on 10 April 2025) was used.

### 2.7. GRADE Assessment

The GRADE methodology was used to assess the certainty of the evidence and the degree of recommendation of the intervention in terms of all outcomes. GRADE was based on its domains, such as risk of bias, inconsistency, indirectness, imprecision, and publication bias, which were some of the evaluated criteria. The certainty of the evidence was determined by the outcome and described in the summary of results (SoF) tables, which were created using the online software GRADEpro GDT (https://www.gradepro.org; accessed on 10 April 2025).

## 3. Results

### 3.1. Selection of Studies

**Figure 1:**
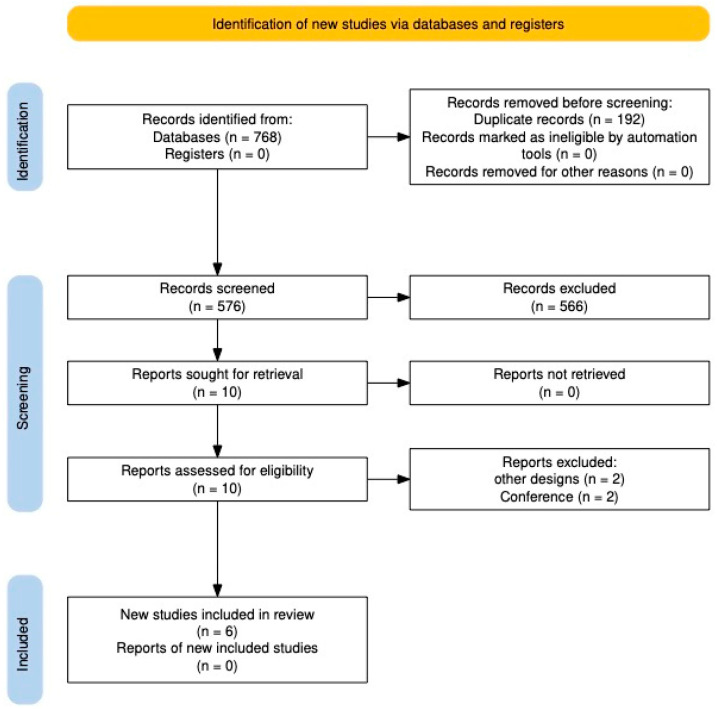
PRISMA flowchart.

After searching the databases, 768 studies were found, of which 192 were eliminated as duplicates. The following 576 studies were evaluated by title and abstract, and 566 were excluded. The remaining studies were reviewed in full text, and four were eliminated because they did not meet the eligibility criteria. Finally, six studies were included in the systematic review [2,6,15,16,17,18] (Figure 1).

### 3.2. Characteristics of Studies Included

The included studies comprised seven clinical trials, each investigating the therapeutic potential of solanezumab across various stages of Alzheimer’s disease and diverse geographic locations. Most trials were multinational in design, encompassing countries such as the United States, France, Japan, Australia, and Spain. Most were randomized, double-blind, placebo-controlled phase 3 trials, with durations of follow-up ranging from 12 weeks to as long as 240 weeks, thereby capturing both short-term and long-term outcomes of solanezumab use (Table 1).

The study populations represented a spectrum of Alzheimer’s disease progression, from preclinical stages to mild and moderate dementia. Inclusion criteria varied across trials but typically required patients to be older adults (ranging from 55 to 90 years), with Mini-Mental State Examination (MMSE) scores spanning 16 to 26, and, in some trials, confirmation of amyloid positivity by PET imaging or cerebrospinal fluid biomarkers. The sample sizes ranged significantly, with the smallest study enrolling 52 participants and the largest including more than 2000 individuals. Across the studies, the proportion of female participants consistently ranged between 53.8% and 59.35%, and the average age of participants was generally in the early seventies, with the notable exception of the dominantly inherited Alzheimer’s disease (DIAD) study, where the mean age was approximately 43 years.

The intervention across trials predominantly involved solanezumab administered at 400 mg every four weeks, although higher doses, such as 1600 mg every four weeks, were used in preclinical populations. One trial utilized a dose-escalation approach in participants with DIAD, gradually increasing the dose from 400 mg to 1600 mg. A notable phase II trial evaluated multiple dosing regimens, including weekly and monthly administrations at 100 and 400 mg, respectively.

Comparison groups across all studies consisted of placebo-controlled arms, and outcomes were assessed through validated cognitive and functional measures. The most utilized tool was the Alzheimer’s Disease Assessment Scale-Cognitive Subscale (ADAS-Cog) in both its 11-item and 14-item forms. Other frequently reported outcomes included the MMSE, the Alzheimer’s Disease Cooperative Study–Activities of Daily Living (ADCS-ADL), the Clinical Dementia Rating–Sum of Boxes (CDR-SB), and disease-specific composite scales such as the Preclinical Alzheimer Cognitive Composite (PACC) and the Dominantly Inherited Alzheimer Network (DIAN) multivariate endpoint. In addition to cognitive measures, several studies evaluated functional abilities, neuroimaging findings (including amyloid PET and ARIA-related MRI abnormalities), and patient- or caregiver-reported quality-of-life metrics.

### 3.3. Effects of Solanezumab on Outcomes

**Figure 2:**
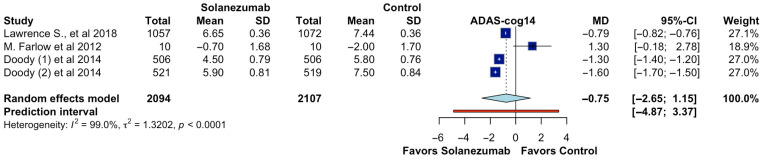
Effect of solanezumab on ADAS-cog14. Doody et al. 2014 [2]; M. Farlow et al. 2012 [18]; Lawrence S. et al. 2018 [6].

In the pooled analysis of four randomized controlled trials reporting ADAS-cog14 outcomes, 4201 participants were evaluated (2094 in the solanezumab group and 2107 in the control group). The overall effect estimate did not demonstrate a statistically significant difference in cognitive performance between solanezumab and placebo. The calculated mean difference (MD) was −0.75 (95% CI: −2.65 to 1.15; *p* > 0.05; CoE Very Low; Figure 2), indicating that the treatment did not meaningfully reduce cognitive decline as measured by the ADAS-cog14 scale. Furthermore, the prediction interval ranged from −4.87 to 3.37, suggesting considerable uncertainty about the effect in future settings. The analysis revealed substantial heterogeneity among the included studies (I^2^ = 99.0%, τ^2^ = 1.3202, *p* < 0.0001), reflecting significant variability in the magnitude and direction of effects across trials. This heterogeneity may stem from differences in sample size, patient characteristics, follow-up durations, or methodological aspects such as dosing protocols or cognitive assessment timing. The individual study estimates showed inconsistent findings. At the same time, some trials favored solanezumab, while others demonstrated either no effect or favored placebo, highlighting the overall inconsistency of the therapeutic benefit across different contexts.

**Figure 3:**
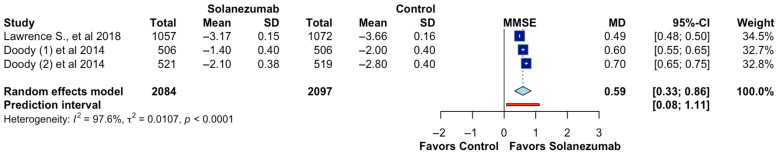
Effect of solanezumab on MMSE. Doody et al. 2014 [2]; Lawrence S. et al. 2018 [6].

Three studies reported data on cognitive function as measured by the Mini-Mental State Examination (MMSE), including 4181 participants (2084 in the solanezumab arm and 2097 in the control arm). The meta-analysis revealed a statistically significant benefit in favor of solanezumab, with a pooled mean difference of 0.59 points (95% CI: 0.33 to 0.86; *p* < 0.05; CoE Moderate; Figure 3), suggesting a modest improvement in cognitive performance compared to placebo. The prediction interval ranged from 0.08 to 1.11, indicating that future studies will also likely detect a favorable effect within this range. However, substantial statistical heterogeneity was observed (I^2^ = 97.6%, τ^2^ = 0.0107, *p* < 0.0001), implying considerable variability across studies. While the direction of the effect remained consistent across the trials, the magnitude of improvement varied, possibly due to differences in baseline cognitive status, duration of treatment, or participant demographics. Despite these limitations, the findings suggest that solanezumab may exert a small but measurable cognitive benefit, as captured by MMSE.

**Figure 4:**
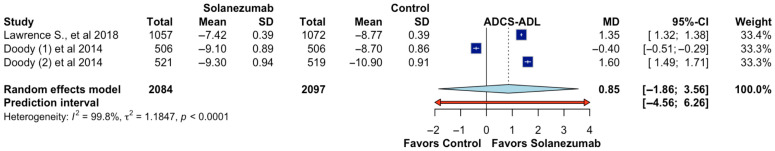
Effect of solanezumab on ADCS-ADL. Doody et al. 2014 [2]; Lawrence S. et al. 2018 [6].

The impact of solanezumab on functional ability, as assessed by the Alzheimer’s Disease Cooperative Study–Activities of Daily Living (ADCS-ADL) scale, was evaluated in three randomized trials comprising a total of 4181 participants (2084 assigned to solanezumab and 2097 to placebo). The pooled mean difference between groups was 0.85 points (95% CI: −1.86 to 3.56; *p* > 0.05; CoE Very Low; Figure 4), indicating no statistically significant improvement in daily functional performance in patients treated with solanezumab compared to placebo. The prediction interval, ranging from −4.56 to 6.26, further reflects wide uncertainty and the potential for null or adverse effects in future research settings. A high level of heterogeneity was observed (I^2^ = 99.8%, τ^2^ = 1.1847, *p* < 0.0001), suggesting substantial inconsistency in the magnitude and direction of effects across the included studies. One trial (Doody et al. [2]) showed a small effect favoring control, while the other two trials favored solanezumab, but the differences were variable and lacked precision. These discrepancies may be attributable to variations in patient functional status at baseline, follow-up duration, or assessment methods used across studies. Overall, the evidence does not support a consistent functional benefit of solanezumab based on the ADCS-ADL measure.

**Figure 5:**
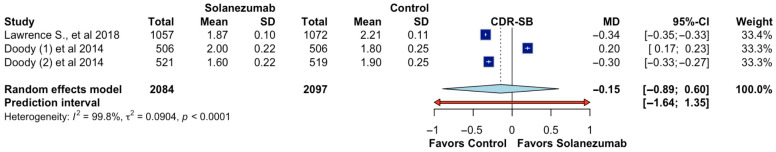
Effect of solanezumab on CDR-SB. Doody et al. 2014 [2]; Lawrence S. et al. 2018 [6].

Three randomized controlled trials assessed the impact of solanezumab on functional and cognitive decline using the Clinical Dementia Rating–Sum of Boxes (CDR-SB) score, encompassing a total of 4181 participants (2084 assigned to solanezumab and 2097 to placebo). The pooled analysis demonstrated no statistically significant difference between treatment and control groups, with a mean difference of −0.15 (95% CI: −0.89 to 0.60; *p* > 0.05; CoE Very low; Figure 5), indicating that solanezumab did not significantly slow disease progression as measured by the CDR-SB scale. The wide prediction interval (−1.64 to 1.35) further underscores the variability in potential effects across future populations and contexts. The heterogeneity among studies was substantial (I^2^ = 99.8%, τ^2^ = 0.0904, *p* < 0.0001), reflecting marked differences in study findings. While one study favored solanezumab modestly, another showed a slight benefit for placebo, and the third revealed nearly neutral effects. These inconsistencies may stem from differences in baseline severity, patient selection, or sensitivity of outcome measurements. These findings suggest that solanezumab does not produce a clinically meaningful improvement in overall functional and cognitive status when assessed through the CDR-SB scale.

### 3.4. Risk of Bias

**Figure 6:**
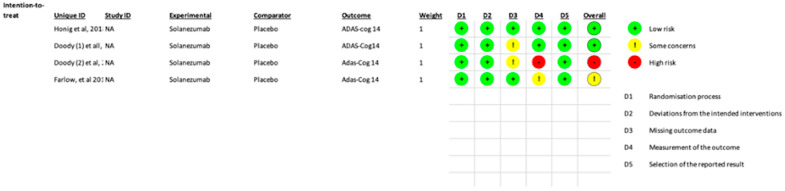
Risk of bias assessment. Doody et al. 2014 [2]; M. Farlow et al. 2012 [18]; Honig Lawrence S. et al. 2018 [6].

Overall, one study had high concerns, and one had some concerns. In the missing outcome data, two studies showed some concerns about bias, and in the measurement of the outcome, one study showed high concern while the other showed some concerns of bias. Doody et al. [2] showed a high risk of bias because of a change in the primary outcome measurement during the middle of the study (from ADAS-Cog 11 to ADAS-Cog14). In the same study, as well as in Doody et al. [2]), there were some concerns of bias in the missing-outcome data domain as there was a significant loss of data (>10%) post-randomization; however, since the losses were similar in both groups, the bias was probably not noteworthy. One study (Farlow et al. [18]) had some concerns about measuring the outcome as the assessment of the outcome in several weeks was likely influenced by knowledge of intervention assignment. Regarding selection, detection, reporting, and other biases, the two trials left showed either low or unclear risk (Figure 6).

## 4. Discussion

This updated systematic review and meta-analysis synthesized data from seven randomized controlled trials to assess the therapeutic efficacy of solanezumab across the Alzheimer’s disease spectrum. While solanezumab showed a small but statistically significant benefit on the MMSE scale, it did not demonstrate meaningful improvements in global cognition (ADAS-cog14), functional ability (ADCS-ADL), or disease severity (CDR-SB). These findings reinforce the growing body of evidence suggesting the limited clinical utility of solanezumab in symptomatic and preclinical Alzheimer’s disease populations.

The lack of benefit observed in ADAS-cog14 aligns with findings from the pivotal EXPEDITION 1 and 2 trials, which reported non-significant differences between solanezumab and placebo in cognitive and functional outcomes over 18 months [17]. Although exploratory analyses within these trials suggested a possible modest benefit among participants with mild AD, these trends were not robust enough to support clinical relevance. The subsequent EXPEDITION 3 trial [6], which specifically targeted individuals with mild dementia and biomarker-confirmed amyloid positivity, confirmed the lack of significant efficacy in this subgroup, with an estimated between-group difference of only −0.80 points on ADAS-cog14 (95% CI: −1.73 to 0.14; *p* = 0.10).

Furthermore, solanezumab failed to prevent or slow cognitive decline in individuals with preclinical Alzheimer’s disease over a 240-week treatment period, as shown in the A4 trial led by Sperling et al. [16]. Despite targeting amyloid-positive individuals without clinical symptoms, the trial did not demonstrate meaningful effects on cognitive composite scores or conversion rates to symptomatic disease. This finding is echoed by the digital cognitive outcome analysis using the Computerized Cognitive Composite (C3), which failed to detect significant benefits [18]. These consistent null results across disease stages suggest that amyloid monomer clearance alone may not sufficiently modify disease trajectory.

In the context of autosomal dominant Alzheimer’s disease, the DIAN-TU-001 trial evaluated solanezumab in genetically predisposed individuals. Results showed no cognitive benefit, even among asymptomatic carriers treated over 4 to 7 years [17]. Participants receiving solanezumab exhibited a numerically more significant decline on some measures than placebo, raising concerns about potential ineffectiveness or even subtle adverse effects in specific subpopulations. Notably, while solanezumab increased plasma and CSF amyloid levels—indicative of peripheral clearance—it failed to produce downstream biomarker effects such as tau or neurofilament light chain modulation, unlike gantenerumab [19].

Although the MMSE analysis in our meta-analysis showed a statistically significant advantage in favor of solanezumab (MD = 0.59; 95% CI: 0.33 to 0.86), this effect size is small and unlikely to reflect meaningful clinical improvement. The high heterogeneity across MMSE results (I^2^ = 97.6%) further limits the generalizability of this finding and suggests variability in trial protocols, populations, and outcome assessments.

These discrepancies raise essential questions about the validity of the amyloid cascade hypothesis as a sole therapeutic target. Solanezumab, which binds soluble monomeric Aβ, may be insufficient to counteract the neurotoxic effects attributed to oligomeric and fibrillar forms or to later-stage pathological cascades such as tau aggregation, neuroinflammation and synaptic degeneration [18]. The repeated clinical failures of solanezumab across disease stages, despite adequate target engagement, underscore the complexity of Alzheimer’s disease pathophysiology and the likely need for combination therapies or multimodal interventions.

This review has several limitations. First, despite including seven major clinical trials, the total number of studies remains limited, reducing the precision of pooled estimates. Second, high statistical heterogeneity was observed across outcomes, which may reflect differences in patient populations, trial designs, dosing regimens, and outcome measurement tools. Third, most studies were conducted in high-income countries, limiting applicability to low- and middle-income settings. Additionally, variations in amyloid positivity confirmation (e.g., PET vs. CSF) and different cognitive composites limit direct comparability across trials. Lastly, some studies changed their primary outcomes mid-trial, raising concerns about selective reporting bias [15].

## 5. Conclusions

Although solanezumab was designed as a disease-modifying therapy targeting soluble amyloid-β, the accumulated evidence from clinical trials has shown limited cognitive benefit in patients with mild-to-moderate Alzheimer’s disease. These findings reflect the ongoing challenges in translating mechanistic hypotheses into effective clinical interventions. While the monoclonal antibody has demonstrated a favorable safety profile, its modest efficacy underscores the need to re-evaluate amyloid-centric approaches. Future therapeutic strategies must adopt a more integrative perspective—targeting tau pathology, neuroinflammation, synaptic dysfunction, and vascular contributions—combined with early diagnosis and personalized interventions. Furthermore, sustained global investment in biomarker infrastructure, clinical trial innovation, and interdisciplinary collaboration will be essential to advance the treatment landscape for this devastating neurodegenerative condition.

## Figures and Tables

**Table 1:** General characteristics of included studies.

Author	[15]	[15]	[16]	[6]	[17]	[2]	[2]	[18]
Country	Multinational	Multinational	Multinational	Multinational	Multinational	Multinational *	Multinational *	USA
Study design **	Phase 3	Phase 3	Phase 3	Phase 3	Observational data, double blind	Observational data, double-blind	Observational data, double-blind	Phase 2
Follow up	80 weeks	80 weeks	240 weeks	80 weeks	200–365 weeks	80 weeks	80 weeks	12–52 weeks
Sample Size	1012 patients	1040 patients	1169 patients	2129 patients	144 patients	1012 patients	1040 patients	52 patients
Baseline diagnosis and severity	Mild to moderate Alzheimer confirmed by MMSE and NINCDS-ADRDA criteria	Mild to moderate Alzheimer confirmed by MMSE and NINCDS-ADRDA criteria	Preclinical Alzheimer	Mild dementia confirmed by MMSE	DIAD *** asymptomatic or mild symptomatic stages	Mild to moderate Alzheimer	Mild to moderate Alzheimer	Mild to moderate Alzheimer
Female gender/age (% and mean + SD)	57.9%/74.7 ± 7.95 years	54.7%/72.45 ± 7.9 years	59.35%/71.95 ± 4.85 years	58.9%/± 7.9 years	43.5 ± 9.875 years	57.9%/74.7 ± 7.95 years	54.7%/72.45 ± 7.9 years	53.8%/71.2 ± 9.2 years
Dose/frecuency	400 mg every 4 weeks	400 mg every 4 weeks	1600 mg every 4 weeks	400 mg every 4 weeks	Dose escalated to target levels	400 mg every 4 weeks	400 mg every 4 weeks	100 mg every 4 weeks, 100 mg weekly, 400 mg every 4 weeks or 400 mg weekly for 12 weeks
Outcomes	ADAS-cog11, ADCS-ADL, MMSE, CDR-SB, NPI, EQ-5D, RUD-Lite, QOL-AD	ADAS-cog11, ADCS-ADL, MMSE, CDR-SB, NPI, EQ-5D, RUD-Lite, QOL-AD	PACC score, CFI, ADL, CDR-SB, amyloid PET	ADAS-cog14, MMSE, ADCS-ADL, CDR-SB, iADRS	DIAN–MCE, CDR-SB, FAS, amyloid PET	ARIA-E and ARIA-H	ARIA-E and ARIA-H	ADAS-cog, safety evaluations, pharmacokinetics, and biomarkers in plasma and cerebrospinal fluid

* Sixteen centers involved; ** randomized placebo controlled; *** dominantly inherited AD.

## Data Availability

Data are contained within the article.

## Supplementary Materials

The following supporting information can be downloaded at https://www.mdpi.com/article/10.3390/pharmaceutics17080999/s1, Table S1: Strategy search.
