# Supplementary material for: The Efficacy of Solanezumab in Patients with Alzheimer’s Disease: A Systematic Review and Meta-Analysis of Clinical Trials
Source: Pharmaceutics. 2025 Jul 31;17(8):999. doi: 10.3390/pharmaceutics17080999 (PMC12389259; doi:10.3390/pharmaceutics17080999)
Supplement: Supplementary file 1 [file pharmaceutics-17-00999-s001.zip › pharmaceutics-3651363-supplementary.pdf]

The Efficacy of Solanezumab in Patients with Alzheimer’s Disease: A Systematic Review and Meta-Analysis of Clinical Trials

Supplementary Table S1. Search strategy.

Engine	Strategy	Results
Pubmed	#1= ("Alzheimer Syndrome" OR "Alzheimer-Type Dementia" OR "Alzheimer Type Dementia" OR "Dementia Alzheimer-Type" OR "Alzheimer's Diseases" OR "Alzheimer Diseases" OR "Alzheimers Diseases" OR "Alzheimer Dementia" OR "Alzheimer Dementias" OR "Dementia, Alzheimer" OR "Alzheimer's Disease" OR "Dementia, Senile" OR "Senile Dementia" OR "Dementia, Alzheimer Type" OR "Alzheimer Type Dementia" OR "Senile Dementia, Alzheimer Type" OR "Alzheimer Type Senile Dementia" OR "Primary Senile Degenerative Dementia" OR "Alzheimer Sclerosis" OR "Sclerosis, Alzheimer" OR "Dementia, Primary Senile Degenerative" OR "Dementia, Presenile" OR "Presenile Dementia" OR "Acute Confusional Senile Dementia" OR "Senile Dementia, Acute Confusional" OR "Alzheimer Disease, Early Onset" OR "Early Onset Alzheimer Disease" OR "Presenile Alzheimer Dementia" OR "Alzheimer Disease, Late Onset" OR "Late Onset Alzheimer Disease" OR "Alzheimer's Disease, Focal Onset" OR "Focal Onset Alzheimer's Disease" OR "Familial Alzheimer Disease" OR "Alzheimer Disease, Familial " OR "Familial Alzheimer Diseases")	63
	#2= (Solanezumab)	
	#3= ("Randomized Controlled Trial" OR "Randomised Controlled Trial" OR "Clinical Trials, Randomized" OR "Trials, Randomized Clinical" OR "Controlled Clinical Trials, Randomized" OR "controlled clinical trial" OR "clinical trial")	
	#4= #1 AND #2 AND #3	
Scopus	#1= TITLE-ABS-KEY ("Alzheimer Syndrome" OR "Alzheimer-Type Dementia" OR "Alzheimer Type Dementia" OR "Dementia Alzheimer-Type" OR "Alzheimer's Diseases" OR "Alzheimer Diseases" OR "Alzheimers Diseases" OR "Alzheimer Dementia" OR "Alzheimer Dementias" OR "Dementia, Alzheimer" OR "Alzheimer's Disease" OR "Dementia, Senile" OR "Senile Dementia" OR "Dementia, Alzheimer Type" OR "Alzheimer Type Dementia" OR "Senile Dementia, Alzheimer Type" OR "Alzheimer Type Senile Dementia" OR "Primary Senile Degenerative Dementia" OR "Alzheimer Sclerosis" OR "Sclerosis, Alzheimer" OR "Dementia, Primary Senile Degenerative" OR "Dementia, Presenile" OR "Presenile Dementia" OR "Acute Confusional Senile Dementia" OR "Senile Dementia, Acute Confusional" OR "Alzheimer Disease, Early	525

	Onset" OR "Early Onset Alzheimer Disease" OR "Presenile Alzheimer Dementia" OR "Alzheimer Disease, Late Onset" OR "Late Onset Alzheimer Disease" OR "Alzheimer's Disease, Focal Onset" OR "Focal Onset Alzheimer's Disease" OR "Familial Alzheimer Disease" OR "Alzheimer Disease, Familial " OR "Familial Alzheimer Diseases")	
	#2= TITLE-ABS-KEY (Solanezumab)	
	#3= TITLE-ABS-KEY(“Randomized Controlled Trial” OR “Randomised Controlled Trial” OR “Clinical Trials, Randomized" OR “Trials, Randomized Clinical” OR “Controlled Clinical Trials, Randomized” OR “controlled clinical trial” OR “clinical trial”)	
Web of Science	#1= ("Alzheimer Syndrome" OR "Alzheimer-Type Dementia" OR "Alzheimer Type Dementia" OR "Dementia Alzheimer-Type" OR "Alzheimer's Diseases" OR "Alzheimer Diseases" OR "Alzheimers Diseases" OR "Alzheimer Dementia" OR "Alzheimer Dementias" OR "Dementia, Alzheimer" OR "Alzheimer's Disease" OR "Dementia, Senile" OR "Senile Dementia" OR "Dementia, Alzheimer Type" OR "Alzheimer Type Dementia" OR "Senile Dementia, Alzheimer Type" OR "Alzheimer Type Senile Dementia" OR "Primary Senile Degenerative Dementia" OR "Alzheimer Sclerosis" OR "Sclerosis, Alzheimer" OR "Dementia, Primary Senile Degenerative" OR "Dementia, Presenile" OR "Presenile Dementia" OR "Acute Confusional Senile Dementia" OR "Senile Dementia, Acute Confusional" OR "Alzheimer Disease, Early Onset" OR "Early Onset Alzheimer Disease" OR "Presenile Alzheimer Dementia" OR "Alzheimer Disease, Late Onset" OR "Late Onset Alzheimer Disease" OR "Alzheimer's Disease, Focal Onset" OR "Focal Onset Alzheimer's Disease" OR "Familial Alzheimer Disease" OR "Alzheimer Disease, Familial " OR "Familial Alzheimer Diseases")	58
	#2= (Solanezumab)	
	#3= (“Randomized Controlled Trial” OR “Randomised Controlled Trial” OR “Clinical Trials, Randomized" OR “Trials, Randomized Clinical” OR “Controlled Clinical Trials, Randomized” OR “controlled clinical trial” OR “clinical trial”)	
Embase	alzheimer disease'/exp OR 'alzheimer disease'	122

	solanezumab'<br>randomized controlled trial'	
	TOTAL SEARCH	768
	DUPLICATES	192
	TOTAL REGISTERS AFTER DUPLICATES REMOVED	576
